# Leveraging Information Technology tools to create cost-effective alternatives: Using Google Sheets as a platform for competitive debate and public speaking tabulation

**DOI:** 10.1371/journal.pone.0332576

**Published:** 2025-09-15

**Authors:** Franco Osei-Wusu, William Asiedu, Donald Yeboah, Shuvajyoti Kar, Siddique Abubakr Muntaka, Joel Kwesi Appiah

**Affiliations:** 1 Department of Information Technology Education, AAMUSTED, Kumasi, Ghana; 2 Department of B.Tech (CSE), Kalinga Institute of Industrial Technology (KIIT) Deemed University, India; 3 School of Information Technology, University of Cincinnati, Ohio, United States of America; University of Lahore - Raiwind Road Campus: The University of Lahore, PAKISTAN

## Abstract

Traditional web-based debate tabulation systems like Tabbycat, offer robust features but often pose high costs and accessibility barriers that limit participation and the smooth organization of events. In this work, we present Tab XYZ, a novel debate and public-speaking tabulation platform built on Google Sheets, as a cost-effective alternative to conventional systems. We deployed Tab XYZ’s cloud-based features like Google Apps Script automation, Google Forms for data input, real-time collaboration, to replicate core functionalities of standard tabulation software without the need for dedicated servers or paid licenses. The proposed system was evaluated in five tournaments constituting a total of 435 participants, and compared against a popular web-based platform on key metrics including setup time, user satisfaction, reliability, and error handling. Results indicate that Tab XYZ eliminated all licensing and hosting costs while achieving user satisfaction scores (overall average 4.7 out of 5) comparable to the conventional system (4.6 out of 5). Tab XYZ also demonstrated robust data security and offline-capable error recovery by leveraging Google’s infrastructure. These findings illustrate a viable pathway to leverage readily available IT tools like spreadsheets and cloud services, to create innovative solutions for specialized domains, avoiding the cost and complexity barriers of traditional approaches.

## 1. Introduction

In many sectors, Information Technology (IT) is used as a tool to improve operational processes. Domains such as healthcare, education, and business have harnessed technologies like data analytics to enhance productivity, foster user experience, and gain competitive advantage [1–3]. In educational settings, extracurricular activities like competitive debates and public speaking events have similarly begun incorporating IT tools to promote fairness and efficient competition management. The debate activity is typically facilitated by tabulation systems that keep track of team performance, generate match pairings (“draws”), record judge feedback, determine rankings, and advance teams through tournament rounds [4]. With growing participation in debate competitions, there is an increasing need for tabulation tools to be highly scalable and optimized for larger pools of teams and judges.

Tabbycat is a web-based open-source system that is used to manage debate tournaments [4]. It is widely used for debate competitions because of its detailed features that meet tabulation demands. Tabbycat is hosted via calicotab.com [4] and supports basic functions like automated draw generation and real time rankings. This web-based application promotes active technology use in extracurriculars and is popular for its support of different debate formats, accessibility and ease of use [5]. But as a web application, Tabbycat has obvious problems related to the category it falls under; web-based systems. As described in [5], web applications have several limitations which include; high dependence on stable internet connections, requires technical expertise for their management, and limited database storage.

In this paper, we introduce Tab XYZ, a lightweight and cost-effective tabulation system built entirely on the Google Sheets platform, as an alternative to conventional web-based debate tabulation software. We demonstrate that leveraging an existing cloud service (Google Sheets) with built-in scripting and database capabilities can replicate the core functionalities of dedicated tabulation systems such as automated team pairing, dynamic ranking calculations, and feedback management, with minimal setup and at no monetary cost. We also present a comprehensive comparative evaluation: we evaluated Tab XYZ side-by-side with the popular system, Tabbycat, using metrics including user satisfaction, ease of setup, system reliability, data security, and error handling. Our study provides evidence that Tab XYZ can achieve comparable performance to the conventional platform on these metrics while eliminating the need for dedicated servers or paid licenses. Collectively, these contributions highlight a novel approach to tournament management, showing how widely accessible tools can be repurposed to address niche operational challenges in education.

The rest of the paper is organized as follows; in Section 2: Literature Review we review the literature on integral concepts at the core of our proposed framework. In Section 3: Methodology we present the methodology for our proposed system. Section 4: Results & Discussion focuses on the analysis of the results presented, and finally, Section 5: Summary & Conclusion provides the Summary and Conclusion of the paper.

## 2. Literature review

### Leveraging IT tools for cost-effective solutions

In academic settings, Information Technology (IT) tools play a crucial role in enhancing both classroom and extracurricular activities. [6] emphasizes that integrating digital tools in education facilitates interactive learning and collaboration, preparing students for modern challenges. [7] posit, that these tools facilitate interactive learning, improve engagement, and support collaborative efforts among students. They explain that integration of IT tools not only enriches the educational experience but also prepares students for modern challenges. More precisely, in the study by [8] the authors present a study on the incorporation of IT tools in activities outside of the classroom but within academic settings. From their findings, they explained that as beneficial as such technologies are, their utilization should also promote cost-effectiveness alongside enhancing productivity or seamless use. Additionally, beyond academia, [9] note that IT tools used in data mining for forecasting production parameters are usually very accurate and improve decision making, but come with a lot of cost components. The authors then made suggestions on areas of focus such as data quality where IT tools could be leveraged to help yield a cost-effective yet productive implementation. The central idea of this review section is to highlight that the principle of maintaining cost-effectiveness while implementing IT solutions in any context, is as important as solving the problem at hand. Hence, a strong motivation for the proposed system in this study.

### Existing web-based tabulation systems

Tabbycat is a web-based platform for debate tabulation as documented in [4]. This conventional web-based system is evidence of integrating Information Technology (IT) into the management of debate. The system is characterized by core functionalities like comprehensive feedback details, draw generation, and live automated rankings. As a web application system, Tabbycat has drawbacks, including manual security configurations due to reliance on external servers. In [10–14] the authors argue that such dependency results in vulnerabilities that cybercriminals can exploit due to the increased likelihood of errors. Also, the requirement for technical expertise in managing web applications is one of their limitations [15–16]. Finally, [17–20] argue that the initial setup as well as the general cost associated with running a web application as key drawbacks to their accessibility especially for smaller or resource-constrained organizations. These general limitations of running web applications like Tabbycat, create the need for an IT solution that employs an alternative platform to address the challenges with similar or better efficiency. This is even more dire in the case of debate tabulation systems because the target individuals or groups of individuals constitute students who require the most accessible and cost-effective approaches in enhancing their extracurricular engagements.

### Google sheets as platform for IT innovations

Google Sheets, part of Google’s cloud-based office suite, has evolved from a simple spreadsheet program into a flexible platform for custom applications. Its built-in scripting capability (Google Apps Script) and interoperability with other Google services allow developers to create specialized solutions on top of a free, hosted infrastructure. [21] highlighted Google Sheets’ scalability, demonstrating that with Apps Script, Sheets can handle surprisingly large datasets and concurrent users, thanks to Google’s cloud backend. [22] similarly showed how a cloud-based application built on Google Sheets can integrate data search functionalities, capitalizing on Google’s reliable uptime and ease of deployment. A study by [23] provides an illustrative example: they developed an HR funding management information system using Google Apps Script within Sheets, achieving automation of complex tasks without a standalone server. One key advantage repeatedly noted in such studies is cost: since Google Sheets is freely accessible and requires no separate hosting, solutions built on it drastically cut down deployment cost [24]. Moreover, Google Sheets supports offline synchronization; [25] discuss how its offline mode on mobile devices can even facilitate learning activities without continuous internet – a feature relevant to reliability. For educational contexts, these characteristics (free access, ease of sharing, real-time collaboration, and offline capability) make Google Sheets a compelling foundation for building custom tools [26]. In summary, Google Sheets provides a versatile, cost-effective platform for creating lightweight applications, motivating our choice to build Tab XYZ on it. We leverage Google Sheets to reproduce and enhance core functionalities of a debate tabulation system, hypothesizing that this approach can meet the needs of debate tournaments with significantly reduced overhead compared to traditional systems.

### Spreadsheet platforms in educational event management

Recent studies highlight the growing use of cloud-based spreadsheets as cost-effective platforms for managing educational events and competitions. For example, educators have leveraged Google Sheets for classroom activities: [26] integrated spreadsheet-driven interactive games into business courses and observed significantly improved learning outcomes compared to a control group, underscoring the platform’s instructional flexibility. Likewise, [24] reports that using Google Sheets to deliver dynamic written feedback in language classes not only enhanced students’ writing performance but also reduced teacher workload. These examples illustrate how spreadsheet-based tools can serve as practical, low-cost alternatives for organizing academic events and exercises, offering ease of use and broad accessibility in educational settings [24,26]. These studies indicate that spreadsheet platforms are becoming invaluable in educational event management; from streamlining the tabulation of scores to enhancing the quality and fairness of performance assessments.

## 3. Methodology

The study employs a mixed-methods approach to evaluate the efficacy and feasibility of Tab XYZ, a Google Sheets-based tabulation system for competitive debate and public speaking. This methodology combines qualitative insights with quantitative data, allowing for a comprehensive analysis of Tab XYZ compared to established platforms like Tabbycat. The following sections outline the key aspects of this investigation.

### System development and design

**Fig 1:**
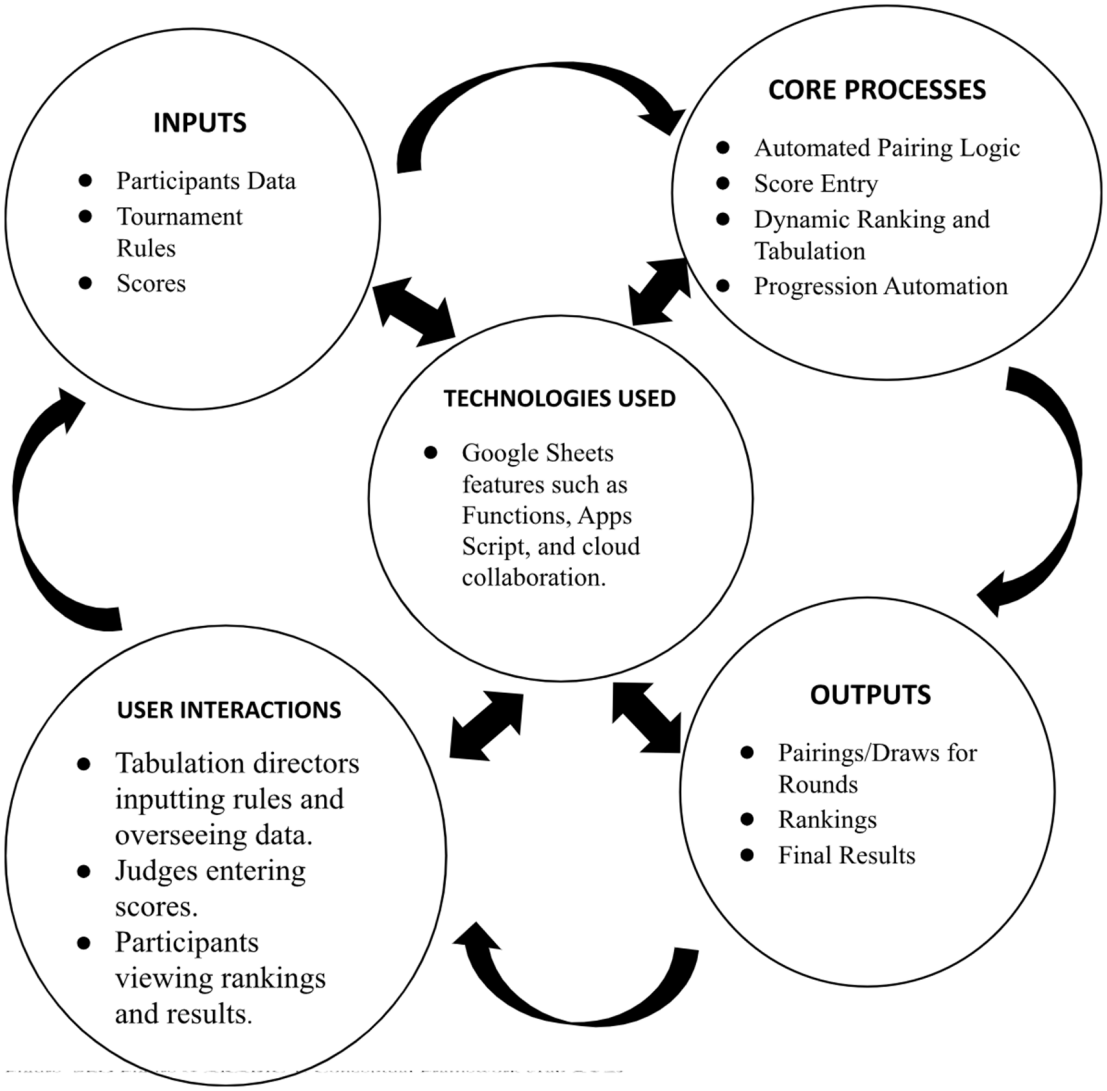
Conceptual framework (Tab XYZ).

Tab XYZ was created in Google Sheets, utilizing its built-in features like formulas, conditional formatting, and data validation. The development prioritized user-friendliness and flexibility while aiming to reproduce core tabulation functionalities found in platforms like Tabbycat. Fig 1 represents the conceptual framework of Tab XYZ, highlighting the connections between the inputs, core processes, outputs technologies used and user interactions. This framework emphasizes how these processes are interwoven to produce an efficiently automated tabulation system for debate and public speaking tournaments (via Google sheets). The inputs mainly include tournament data, participants’ data, feedback scores on speakers as well as feedback scores on judges. Since the inputs’ integrity and accuracy directly affects the effective operationalization of the system, the need for user-friendly interfaces and data collection mechanisms (via Google forms) is central to the development of this framework. The system’s core processes comprise automated pairing logic, dynamic ranking and tabulation, and automated progression. Automated pairings ensure fair and efficient match-ups, minimizing bias and manual errors. Real-time ranking and tabulation provide instant feedback, while automated progression streamlines advancement to subsequent rounds, especially crucial in larger tournaments. By virtue of this automation, the tournament tends to benefit from accurate and seamless management since the workload of manual operations are eliminated. Since the goal of creating this novel tabulation system is to provide a cost-effective and flexible alternative, utilization of in-built Google Sheets features like App Script, Spreadsheet functions, Google Forms, as well as leveraging the platform/s real-time collaboration are paramount. Such flexibility of the Google Sheets platform is the pre-requisite for meeting the customized tournament needs. Another relevant component of the system’s framework which fosters user experience, and promote transparency and accountability, is user interaction. This section of the framework encompasses scores and feedback entries, public viewing of results and rankings via the HTML option in Google Sheets and among others. Finally, the system outputs include pairings/draws, rankings, and final tournament results, dynamically generated and presented in real time, providing timely and accurate information.

### System implementation

**Fig 2:**
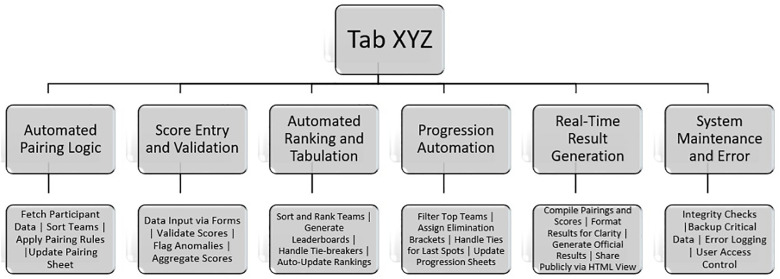
Functional architecture of Tab XYZ.

The proposed framework was implemented to execute six key functionalities; Automated Pairing Logic, Score Entry and Validation, Automated Ranking and Tabulation, Progression Automation, Real-Time Result Generation, and System Maintenance and Error. Fig 2, which illustrates the Functional Architecture of Tab XYZ, details the specific processes categorized under each core function. It is worth noting that the proposed framework is not only limited to these highlighted functionalities but also encompasses auxiliary features. Additionally, the Tab XYZ framework is such that it makes room for scalability of functionalities. This is made possible via the use of Apps Script and Spreadsheet Functions. Features within Google Sheets. This approach provides clarity and conciseness without overlooking the system’s broader capabilities. Fig 2 below illustrates the architecture of the system’s functionalities.

### Scripts of key functionalities

**Fig 3:**
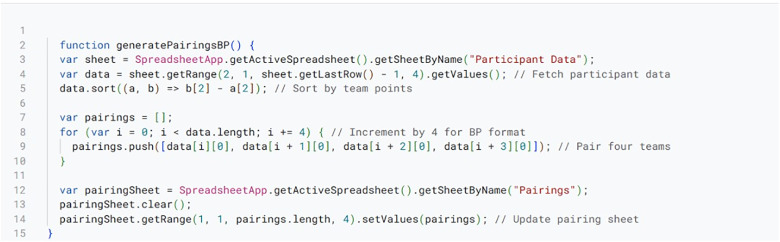
Automated Pairing Script.

**Fig 4:**
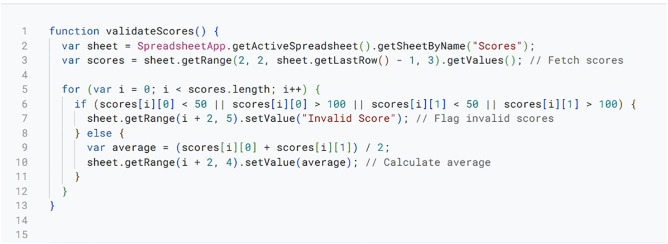
Score Entry and Validation Script.

**Fig 5:**
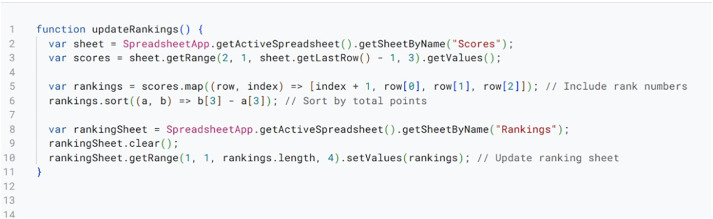
Automated Ranking and Tabulation Script.

**Fig 6:**
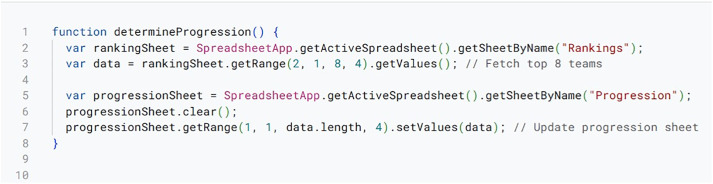
Progression Automation Script.

**Fig 7:**
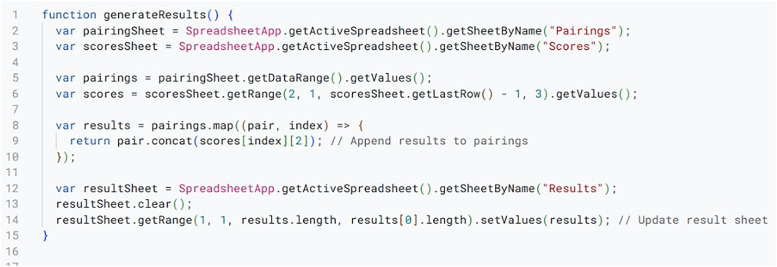
Real-Time Result Generation.

**Fig 8:**
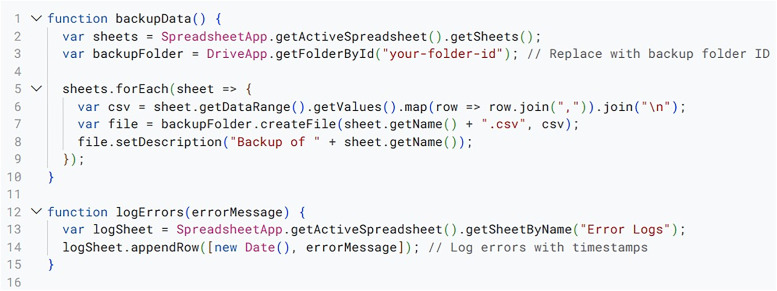
System Maintenance and Error Handling Script.

The Tab XYZ system automates and streamlines debate tournament management through a framework of key functionalities implemented using scripts within Google Sheets. These functionalities are realized through various scripts presented in Figs 3–8, that are embedded within the Google sheets platform.

First, in Fig 3, we present the script used to create the automated and unbiased pairings for each round. Draw generation (through the automated pairing) is one of the most challenging yet very important tasks in a debate and public speaking tournaments. From the script, the system produces either power or random match-ups for participants, depending on tournament rules specified by administrators (conventionally called tab directors [4]). This script retrieves participant data from designated spreadsheet ranges, then employs sorting algorithms to rank teams according to their performance metrics (e.g., team points, speaker scores) in previous rounds. Match-ups are generated by pairing similarly ranked teams, while simultaneously maintaining side balance. The script then populates the pairing sheet with these automatically generated pairings. Executed at the conclusion of each round, this script ensures prompt preparation for the subsequent round and significantly reduces manual errors.

Again, in Fig 4, we present a dedicated script that validates entered scores, confirming that the scores fall within the permitted ranges (e.g., speaker scores between 50 and 100). It flags inconsistencies such as disallowed ties in winning points for user correction. Speaker averages and team points totals are calculated automatically and validated scores are recorded in a central sheet for processing later. When judges submit scores, they undergo validation and storage for use in rankings and progression calculations. The Tab XYZ system incorporates this rigorous score entry and validation process to guarantee data accuracy. By automating these functions, the script minimizes the potential errors that could jeopardize the tournament’s fairness. This is important because maintaining tournament integrity hinges on accurate scorekeeping.

In Fig 5, the Tab XYZ system updates participant rankings after each round. This includes team rankings and individual speaker standings updates. This ranking/tabulation script sorts teams by cumulative points, speaker scores and required tie-breaking criteria. Also, it produces leaderboards for team and individual speaker rankings. Tie-breakers are handled by applying secondary metrics such as average speaker points or opposition strength. The script updates the ranking sheets for all parties. This real time updating functionality provides transparency and user satisfaction.

Also see Fig 6 for how Tab XYZ runs Progression Automation to identify qualifying teams based on predefined rules. The progression script filters teams based on cumulative rankings at the end of preliminary rounds. It then pools positions in elimination brackets to ensure even match-ups. The script also handles edge cases like ties for the last advancing positions using additional tie-breaking criteria. Last but not least, it updates progression sheets with list of qualified teams for the next competition phase. This automatically transitions players from preliminary to elimination rounds without disrupting tournament pace and structure.

Generating results such as pairings, rankings, and final outcomes, is a key output functionality of the system. As seen in script presented in Fig 7, data from the pairing, score, and ranking sheets is pulled by a result generation script, which then formats this data into easy-to-read tables, ensuring clarity and accessibility for users. Shared sheets are automatically updated with these results, and PDFs of the official results are also generated. To highlight important details like breaking teams or top speakers, the script incorporates conditional formatting. By providing clear and timely information to all participants, this feature enhances the user experience. These outputs are automatically updated and made accessible to stakeholders via shared sheets.

Finally, in Fig 8, we present a proactive maintenance script which is incorporated into the Tab XYZ system to ensure its smooth and reliable operation. This script addresses potential issues by performing periodic data integrity checks, hence verifying the consistency and accuracy of data within the spreadsheets. Such functionality contributes to the robustness of the Tab XYZ system, ensuring its resilience even in the face of unexpected problems. It does this by logging errors and generating notifications, alerting tabulation directors (administrators) on issues requiring corrective action. Finally, to safeguard against data loss, the script (in Fig 8) periodically back up critical data in Google Drive.

### Implementation of spreadsheet functions

In addition to utilizing Google Apps Script for the implementation, the proposed system also employs Spreadsheet functions in instances that demand niched IT solutions within tabulation management. These Spreadsheet Functions are largely implemented to focus on tasks like access control, automated calculations, and data validation. Some of these Functions include; the VLOOKUP and SORT, used to support the automated ranking to provide real-time tabulation, the ISERROR and COUNTIF functions used for error handling, and the RANK and IF functions are used in augmenting the Automated Pairing Logic script. This combined approach leverages the strengths of both scripting and built-in functions for a robust and highly flexible system. By combining Spreadsheet Functions and the use of App Scripts, the proposed framework takes advantage of their strengths to enhance flexibility and customization.

### Performance evaluation metrics

#### User evaluation and feedback.

The participants involved various individuals who play key roles typical in debate tournaments: tabulation directors (Tab Directors), adjudicators (Judges), speakers (debaters), and observers. We documented basic demographic information, including each participant’s self-reported computer literacy level (categorized as Low, Intermediate, or High). Table 1 *presents the demographic breakdown of the participant pool.* A total of 435 individuals took part: 11 Tab Directors, 98 Judges, 316 Speakers, and 10 Observers. As seen in Table 1, about 77% of users rated themselves as having intermediate computer literacy, 17% high literacy, and 6% low literacy. This indicates that while most users had moderate technical proficiency, a non-trivial minority might rely on a very user-friendly system – underlining the importance of our design emphasis on simplicity and accessibility.

**Table 1:** Demography of Users.

Users	Computer literacy			Total
	Low	Intermediate	High	
Tab Directors	0	5	6	11
Judges	7	69	22	98
Speakers	19	250	47	316
Observers	1	8	1	10
Total	27 (6%)	333(77%)	76(17%)	435

#### Cost-effectiveness and system reliability.

This metric assesses the financial and operational sustainability of Tab XYZ in comparison to traditional web-based tabulation systems like Tabbycat. By utilizing Google Sheets, Tab XYZ eliminates expenses associated with server hosting fees, subscription costs, and complex technical infrastructure. Its reliability is further bolstered by integration with Google’s cloud platform, which ensures consistent performance and minimizes downtime. A comparative cost analysis of setting up and maintaining both systems across various tournaments could be included in this evaluation.

#### Ease of access and setup.

This metric assesses the user-friendliness of the Tab XYZ system, both during initial setup and in daily operation. Leveraging pre-designed templates, Google Forms, and Apps Script, Tab XYZ simplifies tournament management. The evaluation incorporates feedback from tab directors regarding the ease of configuring pairing rules, integrating scores, and managing team progression, specifically focusing on the system’s accessibility for users without advanced technical expertise.

#### Data security and privacy control.

This metric assesses the strength of Tab XYZ’s data protection mechanisms. Several key features contribute to this security: restricted access to the system’s backend for administrators, granular control over user collaboration permissions, and the utilization of Google Sheets’ built-in data validation and protection tools. While transparency is maintained through a public HTML view, sensitive information remains secure, as this view restricts direct access to the underlying data. The evaluation focuses on the system’s effectiveness in preventing unauthorized edits and safeguarding sensitive information.

#### Error handling and recovery.

This metric evaluates the robustness of Tab XYZ’s data protection mechanisms. The system employs several key security features, including restricted backend access for administrators, granular control over collaboration permissions, and utilization of Google Sheets’ built-in data validation and protection tools. Public access is limited to an HTML view, ensuring transparency while safeguarding sensitive information. The evaluation centers on the system’s effectiveness in preventing unauthorized data modification and protecting sensitive information.

## 4. Results & discussion

### Results

#### Participants demographics.

**Fig 9:**
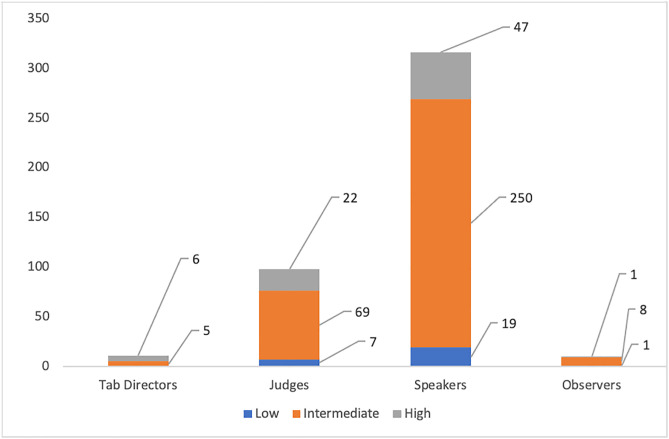
Computer Literacy Levels of Users.

**Fig 10:**
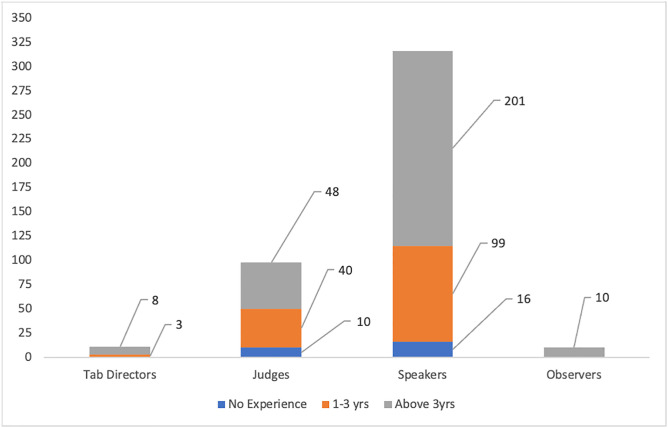
Level of User Experience with Conventional Web-based Tabulation Systems (Tabbycat).

The demography used to test the system was dominated by an approximate 77% of individuals with an intermediate level of computer literacy. This statistic was necessary to inform the ease of use of the Tab XYZ system. Fig 9 provides a detailed visual view of the breakdown of user roles and their computer literacy levels. It is important to note that from Fig 10, approximately 92% of the 435 have experienced the use of conventional web-based tabulation systems like Tabbycat for at least a year. This goes to show that the data collected from the pool of users we engaged for this study are valid to serve as a basis for comparative analysis between our proposed systems and conventional web applications like Tabbycat.

#### User satisfaction metrics.

**Fig 11:**
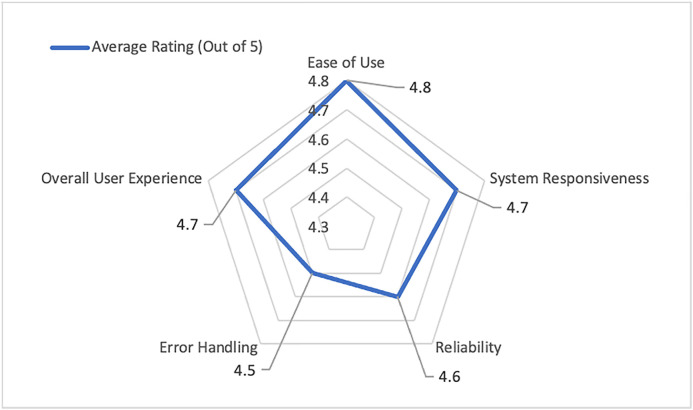
User Satisfaction Scores.

Participants evaluated Tab XYZ based on; Ease of Use, System Responsiveness, Reliability, Error Handling, Overall User Experience on a scale of 0 (lowest) to 5 (highest). Fig 11 shows the average rating by the 435 users based the 5 metrics (S1 Table).

*Ease of Use* received an average rating of 4.8 out of 5. This affirms that the was familiar and straightforward. Judges found the digital ballot via widely used Google Form easier than paper ballots or other software interfaces they had used. A few less tech-savvy users (those in the “Low” computer literacy category) initially expressed nervousness about using a cloud-based tool, but ultimately, they reported little trouble. The *System Responsiveness* metric averaged 4.7. Agreeably, participants noted that updates like pairings and standings were nearly instantaneous after each round, which resulted in such high rating. Some minor points that likely prevented a “5” from every user could include occasional form submission lags which is likely due to local internet speed, which were rare and not systemic. *Reliability* was rated around 4.6. This implies that users generally found the system reliable. Even though the system as a whole did not fail, the slight dip of the *reliability* rating compared to other scores could be attributed to one or two instances where a user experienced a brief connectivity problem, which may have influenced their rating. We also suspect that because Tab XYZ was new, a few users were cautious in giving a perfect reliability score without a longer track record, unlike some who trust long-established systems. *Error Handling* received an average of 4.5, the lowest among the metrics but still positive. Tab staff observed that the automated validation caught most errors, and they appreciated the clear error messages. The reason this score is not higher might be because when for example a judge input an invalid score that got flagged, it required the tab director to intervene and correct it manually. Some tab directors most likely saw this extra step as slightly inconvenient. *Overall Experience* was very high at 4.7. This goes to show that qualitatively, participants were impressed that such a powerful solution could be implemented with Google Sheets. Judges and debaters repeatedly mentioned the convenience of getting immediate results on their phones via the published link, and tab directors highlighted the stress reduction from not worrying about server crashes.

#### Cost-effectiveness & system reliability.

From a cost perspective, Tab XYZ did not incur any additional monetary cost as highlighted in Table 2. All components used (Google Sheets, Forms, Apps Script) are free services. In contrast, to use Tabbycat for the same events, the organizers would have had to either rent a cloud server or dedicate an existing machine and technical support to run it. While these costs are not huge, for small clubs even minor expenses or the need for a tech-savvy volunteer can be prohibitive. The cost-effectiveness of Tab XYZ is thus evident by eliminating major expenses typically associated with traditional web-hosted tabulation systems like Tabbycat. Its reliance on Google Sheets’ free infrastructure, minimal bandwidth requirements, and user-friendly design democratizes access to sophisticated tabulation capabilities, especially beneficial in resource-constrained environments. The comparison in Table 2 demonstrates the transformative potential of leveraging readily available tools for specialized applications like debate tabulation. Additionally, our proposed system poses more reliability due to the elimination of a downtime as a result the competitive advantage under cost metrics such as, internet bandwidth and system maintenance.

**Table 2:** Comparative Analysis on Cost-Effectiveness between Tab XYZ and Conventional Web Applications.

Cost Component	Conventional Web Applications (i.e. Tabbycat)	Tab XYZ
Initial Setup	They require costly processes like server configuration, SSL certification and name registration [17–20]	Uses free Google Sheets templates and tools with no additional software or hardware needed. Hence, minimal setup cost.
Hosting and Infrastructure	Requires a server or cloud hosting service, such as AWS, Google Cloud, or a managed hosting provider that comes at a cost. [17–20]	No cost of hosting because the system operates entirely within the Google ecosystem.
System Maintenance	Regular security patches, updates, and backups, which the hosting provider manages are required. [17–20]	Google Sheets’ infrastructure handles updates and Backups, therefore does not require any direct maintenance.
Customization Costs	Programming expertise is required for advanced customization.	Easy Customization with Google Sheets functions and Apps Script.
Internet Bandwidth	Web applications require high-speed and stable internet for real-time operations. This hugely affects costs in areas with limited connectivity [27,28].	With the availability of a synchronization feature, system can function offline and be updated when connectivity is available. It uses minimal bandwidth.
Training and Onboarding	They usually require users to have an enormous amount of training to understand the interface and workflow. Training is usually costly	Users familiar with Google Sheets require no additional onboarding. Generally, training time is minimal due to the simple interfaces and huge automations in processes like uploading tournament data.
Total Estimated Cost per Tournament	The total estimated cost per tournament is relatively high	Near zero costs.

### Ease of setup

A core principle within Tab XYZ’s framework is user-friendliness. This extends to ensuring a streamlined setup process for tabulation directors by prioritizing simplicity and automation by implementing App Scripts in Google Sheets. Arguably, Tab XYZ is more convenient for tab directors with low levels of expertise due to the leverage of pre-defined templates for various tournament formats (e.g British Parliamentary Debate, Public Speaking etc.). Other than manually inputting details of participants (speakers and judges), or using a third-party application for such task, like what is done in conventional tabulation systems, automatically configures participant names, team allocations, and initial pairings Tab XYZ, using automated scripts to extract participants’ data directly from Google forms. This also enhances time efficiency as little to no manual intervention is required in the setup. On the other hand, Tabbycat provides a detailed set of features for configuring the tabulation setup. This widely used web-based application involves processes like creating.csv files formatted in accordance with the system’s data structure or using third-party applications like Tabtastic [29] for its setup. These features present some drawbacks in Tabbycat’s setup process; it tends to be time-consuming, adds extra layers of cost and requires advanced technical expertise. Manual data entry is often a potentially time-consuming and error-prone process for less experienced users. Additionally, Tab XYZ benefits from Google Sheets’ inherent collaborative features, enabling multiple administrators (tab directors) to work concurrently on setup tasks like configuring pairings, scoring rules, and progression criteria. Meanwhile, Tabbycat’s centralized dashboard, while offering structured oversight, may limit collaborative flexibility. While Tabbycat’s structured setup and detailed configuration options offer robustness and scalability suitable for large, technically proficient tournaments, Tab XYZ prioritizes ease of use and accessibility for a broader range of users. This comparison highlights a trade-off; Tab XYZ emphasizes simplicity and efficiency, while Tabbycat caters to tournaments with the resources to manage a more intricate setup process.

**Fig 12:**
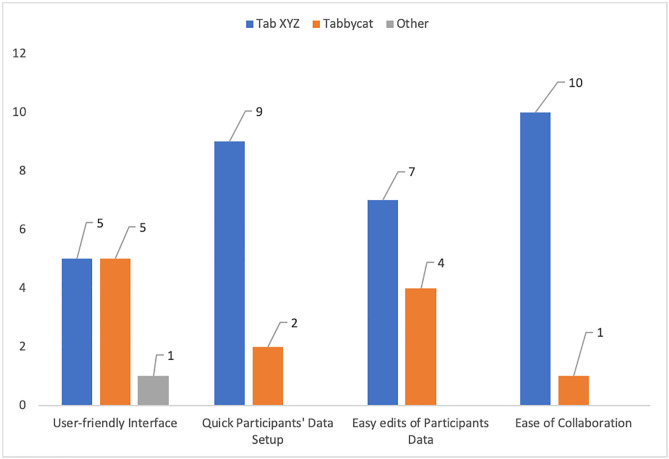
Ease of Setting Up Debate Tabulation Systems.

Fig 12 illustrates responses from the eleven (11) tab directors on the ease of setting up tabulation systems. The responses show that most tab directors find tab XYZ a more convenient tabulation system for setting up tournaments, editing participants’ data, and enhancing collaboration. An equal number of individuals believed that Tab XYZ and Tabbycat have user-friendly interfaces. Because the development of Tab XYZ’s interface is less costly as compared to Tabbycat, having an equal number of respondents underpins the proposed system’s cost-effectiveness.

The high regard for Tab XYZ’s ease of setup (by tab directors) is fairly due to its familiar interface (spreadsheets), which in turn requires minimal training. Tab staff mostly needed to learn how to execute the pairing script and monitor the Sheets, which is arguably straightforward given their existing spreadsheet knowledge. This low learning curve can also be considered a “cost” saving in terms of time and effort.

### Data security and privacy

In tabulation systems, when handling sensitive data like participant information, scores, and feedback, data security and privacy are paramount [30]. A comparison of Tab XYZ and Tabbycat reveals stark differences in their security approaches, stemming from their distinct architectures. Tabbycat, like all other web-hosted systems, relies on external servers, introducing inherent vulnerabilities [30]. While offering advanced features, its security hinges on the hosting environment’s encryption, firewall, and patching, all requiring manual configuration by administrators, and increasing oversight risks [31–32]. Furthermore, securing sensitive data necessitates complex authentication layers, prone to errors if mismanaged [30–32]. Reliance on external hosting also increases Tabbycat’s vulnerability to data breaches, especially if SSL certificates are misconfigured or outdated, particularly during data transmission [31]. Conversely, Tab XYZ leverages Google Sheets’ robust, Google-managed security infrastructure. As explained in [33] such robust security is through granular access controls, the end-to-end encryption features, and multi-factor authentication features. Table 3 presents a concise comparison between the widely used Tabbycat and our proposed Tab XYZ on various aspects of Data Security and Privacy.

**Table 3:** Comparative Analysis on Data Security and Privacy between Tab XYZ and Tabbycat.

Aspect	Tabbycat	Tab XYZ
Encryption	Dependent on the Hosting Provider and requires configuring SSL certificates manually	Automatically Google-managed end-to-end encryption of data
Privacy Settings	Configuration prone to mismanagement also requires technical expertise	Interfaces for managing permissions and controlling data visibility are user-friendly.
Access Control	Custom configuration is required and hence complexity increases as users and roles increase.	Google sheets’ in-built granular access control simplifies access control.
Offline Security	It does not support offline security as all operations require only real-time connection.	Offline data entry with secure synchronization when connection is restored, is supported.

**Fig 13:**
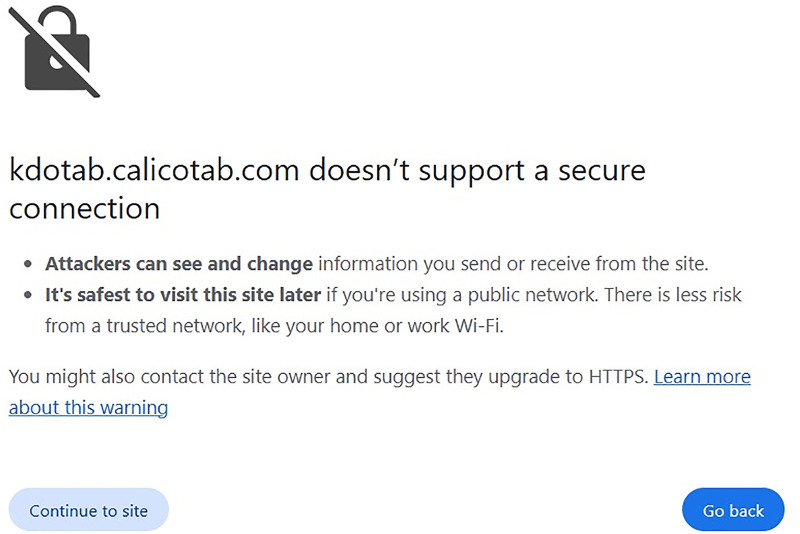
An instance of susceptibility of Web applications (Tabbycat).

Finally, in Fig 13 we present evidence of the likely security vulnerability web applications like Tabbycat are exposed to. Such vulnerability usually come as a result of an oversight or errors due to mismanagement. This is proof to the earlier discussions on the need for regular manual configurations in the case of web-based systems. The problem with such manual security configurations is that it increases the likelihood of error occurrences like what Fig 13 illustrates; a case of bot updating the HTTP with a Secure Socket Layer (SSL). Tab XYZ is free of such limitations due to the Google infrastructure it utilizes, which uses HTTPS along with very robust encryption. Without requiring any updates from users, Google’s security infrastructure conceals data from unauthorized and unauthenticated access, and automatically fixes security patches regularly with no user intervention. These inherent measures ensures that Tab XYZ is always up-to-date regarding security configurations and hence eliminates the risk of vulnerabilities.

### Error handling and recovery

**Fig 14:**
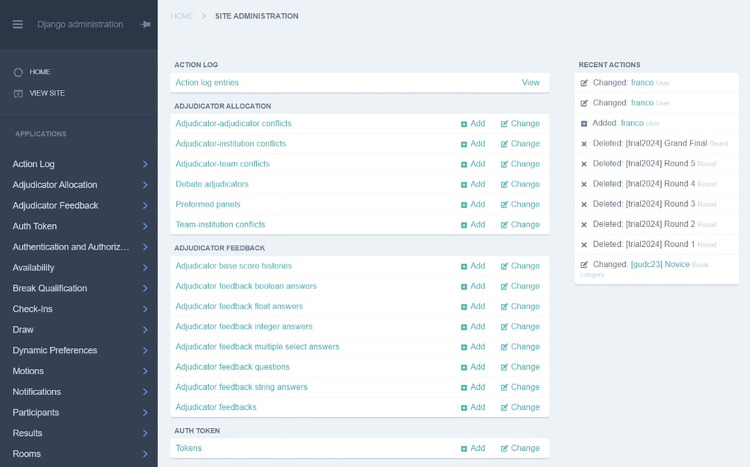
Backend of a Web Application for Debate Tabulation (Tabbycat).

**Fig 15:**
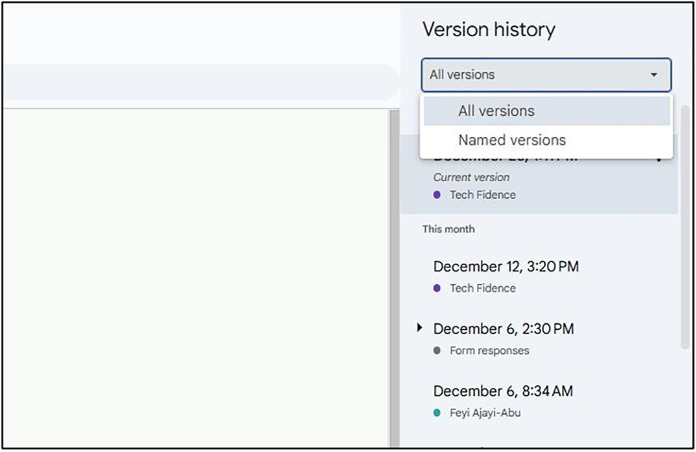
User-Friendly Rollback Options in Tab XYZ via Google Sheets.

**Fig 16:**
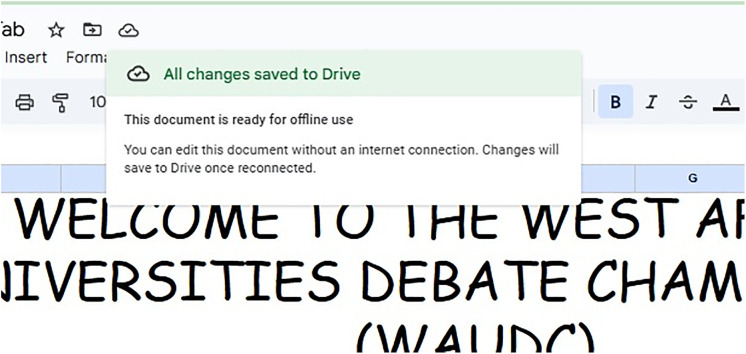
Tab XYZ’s offline synchronization feature via Google Sheets.

Due to the general feature-rich nature of the Tabbycat system, its error-logging and debugging tools are quite advanced and hence characterized by sophistication in highlighting and resolving errors as evident in [4]. As identified regarding most of its operationalization, Tabbycat’s error handling feature used for addressing server downtime, issues from unexpected input formats, incorrectly configured settings, requires a user to have technical expertise to be able to utilize it. Usually, in Tabbycat, either a server host is contacted or backend logs are to be accessed to diagnose and provide solutions to a problem. Additionally, such error rectification is hugely dependent on manual error-prone interventions by users. The interface in Fig 14 is evidence of the complex nature error-handling in Tabbycat while Fig 15 is an indication of our proposed system’s rollback ability. Tab XYZ adopts an offline synchronization feature via Google sheets that minimizes data loss during network interruption. Fig 16 illustrates this. On the side of web-application, the reliance on external serves could cause problems due to uncontrolled server outage. Recovery in such instances is contingent upon the hosting provider’s response time, which can be highly variable. In contrast, Tab XYZ benefits from Google’s globally distributed infrastructure, ensuring high availability and redundancy. Even though no major outages occurred during the tournaments, Figs 15 and 16 show Google’s infrastructure had 100% uptime during our events. Assuming a judge briefly lost internet connectivity while submitting a ballot, due to Google Forms’ tolerance for connectivity loss and offline editing capabilities of Sheets as explained earlier, no data would be lost, and the ballot would be synced once the connection returned. The maintenance or back-up script in Google sheets ran at the end of each round, creating snapshots of results. Having these backups give organizers confidence and peace of mind.

## Discussion

The results indicate that our approach is viable: a spreadsheet-based system can indeed handle the demands of a real tournament and satisfy users. This finding expands the notion of what tools can be used in operational contexts. We found that by building on a robust, widely available platform (Google Sheets), we inherited many benefits: reliability, scalability, and familiarity, without directly writing those functionalities from scratch. Despite our novel system just leveraging Google sheets functionalities, a high user satisfaction was recorded. Contrary to conventional notions that suggest that people prefer sophistications, the feedback suggests that what users care about most are functionality and timeliness, not the underlying technology. As long as the interfaces presented was clear and results were prompt, users were happy. In fact, the feedback via ratings implied that using a Google Form was easier and simpler. The cost-benefit is also noteworthy from an organizer’s perspective. Results presented, especially on the cost effectiveness of Tab XYZ, also emphasize that lowering barriers can enable more events or broader participation. Furthermore, since the deployment of this novel tabulation system relied on Google’s secure platform it benefits from robust and automated security mechanisms, unlike conventional web-based systems that require manual security maintenance. Additionally, results indicated that Google Sheet’s error handling and recovery feature informed high ratings on reliability and error handling from users Agreeably, there are limitations and trade-offs to acknowledge. Google Sheets, while powerful, has some constraints such as script execution quotas. We did not hit those limits in our use, but extremely large-scale tournaments might approach them. Also, while our solution covers the major needs, it is less feature-rich than something like Tabbycat. For instance, Tabbycat supports multiple debate formats and intricate judge assignment algorithms. Tab XYZ, in its current form, was configured for the British Parliamentary format with adjustable points; adapting it to a very different format might require further development.

## 5. Summary & conclusion

In this study, we introduced Tab XYZ, a lightweight and cost-effective alternative to conventional web-based tabulation systems for competitive debate and public speaking. Tab XYZ leveraged the Google Sheets platform and its built-in scripting capabilities to implement key functionalities of tournament tabulation which include automated team pairings, real-time ranking updates, progression of teams, and robust error checking, all within a familiar spreadsheet interface. We evaluated the system through deployment in five real tournaments, comparing its performance and user acceptance to those of a widely used system (Tabbycat) on several metrics: user satisfaction, ease of setup, cost-effectiveness, reliability, data security, and error handling. The results showed that Tab XYZ performed excellently: it provided an intuitive user experience (4.8 out 5 on average) and proved to be highly efficient in operation. Users rated their overall satisfaction with Tab XYZ as very high (4.7 out of 5 on average), essentially on par with the conventional platform. This indicated that the new system did not compromise the competition experience. Notably, Tab XYZ eliminated the infrastructure and licensing costs associated with typical solutions, since it ran entirely on free cloud services. By relying on Google’s secure and resilient infrastructure, the system also inherited strong reliability and data security, addressing common issues found in self-hosted web applications. In summary, by prioritizing flexibility, simplicity, and cost-effectiveness in the design of Tab XYZ, this study demonstrated a practical and innovative way to use readily available IT tools to meet the needs of debate tournament management.

For future work, first, we recommend exploring the development of a more polished user interface to further improve user experience, especially for participants who might prefer a single app-like portal for all tournament information. Second, integrating emerging technologies such as machine learning could provide advanced features. For instance, an ML algorithm could analyze judges’ written feedback to identify common areas of improvement for speakers, or predict breaking teams which could be useful for adjudication core decisions. Third, future research can focus on the scalability of this approach for larger tournaments or different formats, and stress-testing the system with more participants or additional concurrent events to help map its limits. Finally, we see potential in applying the principles of Tab XYZ to other domains that requires coordinated data management and automation like school competitions, and small conferences. These might benefit from a similar Sheets-based solution. We hope that this work inspires further innovations that leverage ubiquitous technologies in creative ways to promote inclusivity and efficiency in educational and extracurricular contexts.

## Supporting information

S1 Table Ratings of respondents on the respective user satisfaction metrics. (PDF)
