# Supplementary material for: Leveraging Information Technology tools to create cost-effective alternatives: Using Google Sheets as a platform for competitive debate and public speaking tabulation
Source: PLoS One. 2025 Sep 15;20(9):e0332576. doi: 10.1371/journal.pone.0332576 (PMC12435689; doi:10.1371/journal.pone.0332576)
Supplement: S1 Table — (PDF) [file pone.0332576.s001.pdf]

S1 Table. Ratings of Respondents on the respective User Satisfaction Metrics

Respondents	Ratings				
	Ease of Use	Error Handling	System Responsiveness	Reliability	Overall User Experience
Respondent 1	5	4	5	4	5
Respondent 2	5	4	4	4	5
Respondent 3	5	5	5	4	5
Respondent 4	5	5	5	5	5
Respondent 5	5	4	4	4	5
Respondent 6	5	5	4	4	5
Respondent 7	4	4	4	3	4
Respondent 8	5	5	5	4	5
Respondent 9	5	4	4	4	4
Respondent 10	5	5	5	5	5
Respondent 11	5	5	5	5	5
Respondent 12	5	4	4	3	5
Respondent 13	5	5	5	5	5
Respondent 14	5	5	5	5	5
Respondent 15	5	4	5	4	5
Respondent 16	5	4	5	4	5
Respondent 17	5	4	4	4	5
Respondent 18	5	5	5	5	5
Respondent 19	4	4	4	4	4
Respondent 20	5	5	5	5	5
Respondent 21	5	4	4	5	5

**Respondent 22**	5	5	4	5	5
**Respondent 23**	5	5	5	4	5
**Respondent 24**	5	4	4	5	5
**Respondent 25**	5	4	5	5	5
**Respondent 26**	5	4	5	5	5
**Respondent 27**	5	4	4	5	5
**Respondent 28**	5	5	5	4	5
**Respondent 29**	5	5	5	5	5
**Respondent 30**	5	4	4	3	5
**Respondent 31**	5	5	5	4	5
**Respondent 32**	5	4	5	5	5
**Respondent 33**	5	4	4	5	5
**Respondent 34**	5	5	5	5	5
**Respondent 35**	5	5	5	5	5
**Respondent 36**	5	4	5	5	5
**Respondent 37**	5	4	4	5	5
**Respondent 38**	5	5	5	5	5
**Respondent 39**	5	5	5	4	5
**Respondent 40**	5	4	5	5	5
**Respondent 41**	5	4	4	5	5
**Respondent 42**	5	5	5	5	5
**Respondent 43**	5	5	5	5	5
**Respondent 44**	5	4	4	4	4

**Respondent 45**	5	5	5	4	5
**Respondent 46**	5	5	5	4	5
**Respondent 47**	5	4	5	5	5
**Respondent 48**	5	4	4	5	5
**Respondent 49**	5	5	5	5	5
**Respondent 50**	5	5	5	5	5
**Respondent 51**	5	4	5	5	5
**Respondent 52**	5	4	4	3	5
**Respondent 53**	5	5	5	4	5
**Respondent 54**	5	5	5	5	5
**Respondent 55**	5	4	5	5	5
**Respondent 56**	5	5	4	5	5
**Respondent 57**	5	5	5	5	5
**Respondent 58**	5	4	5	5	5
**Respondent 59**	5	4	4	3	5
**Respondent 60**	5	4	5	5	5
**Respondent 61**	5	5	5	4	5
**Respondent 62**	5	4	5	5	5
**Respondent 63**	5	4	4	5	5
**Respondent 64**	5	5	5	4	5
**Respondent 65**	5	5	5	4	5
**Respondent 66**	5	4	5	5	5
**Respondent 67**	5	4	4	4	4

**Respondent 68**	5	5	5	5	5
**Respondent 69**	5	5	5	5	5
**Respondent 70**	5	4	5	5	5
**Respondent 71**	5	4	4	5	5
**Respondent 72**	5	5	5	4	5
**Respondent 73**	5	5	5	4	5
**Respondent 74**	5	4	5	4	5
**Respondent 75**	5	4	4	3	5
**Respondent 76**	5	4	5	4	5
**Respondent 77**	5	4	4	5	5
**Respondent 78**	5	5	5	5	5
**Respondent 79**	5	5	5	5	5
**Respondent 80**	5	4	5	5	5
**Respondent 81**	5	4	4	4	5
**Respondent 82**	5	5	5	5	5
**Respondent 83**	5	5	5	5	5
**Respondent 84**	5	4	5	5	5
**Respondent 85**	5	4	4	4	5
**Respondent 86**	5	5	5	4	5
**Respondent 87**	5	5	5	3	5
**Respondent 88**	5	4	5	4	5
**Respondent 89**	5	4	4	3	5
**Respondent 90**	5	5	5	4	5

**Respondent 91**	5	5	5	5	5
**Respondent 92**	5	4	4	5	5
**Respondent 93**	5	5	5	5	5
**Respondent 94**	5	4	5	5	5
**Respondent 95**	5	4	4	5	5
**Respondent 96**	5	5	5	5	5
**Respondent 97**	5	5	5	5	4
**Respondent 98**	5	4	5	5	4
**Respondent 99**	5	4	4	4	5
**Respondent 100**	5	5	5	4	5
**Respondent 101**	5	5	5	4	5
**Respondent 102**	5	4	5	4	5
**Respondent 103**	5	4	4	5	5
**Respondent 104**	5	5	5	5	3
**Respondent 105**	5	5	5	5	5
**Respondent 106**	5	4	5	4	5
**Respondent 107**	5	4	4	3	4
**Respondent 108**	5	5	5	5	5
**Respondent 109**	5	5	5	5	5
**Respondent 110**	5	4	5	5	5
**Respondent 111**	5	4	4	5	4
**Respondent 112**	5	5	5	5	5
**Respondent 113**	5	5	5	5	5

**Respondent 114**	5	4	5	5	5
**Respondent 115**	5	4	4	5	5
**Respondent 116**	5	5	5	4	5
**Respondent 117**	5	5	5	5	4
**Respondent 118**	5	4	5	5	5
**Respondent 119**	5	4	4	5	5
**Respondent 120**	5	5	5	5	5
**Respondent 121**	5	5	5	5	5
**Respondent 122**	5	4	5	4	5
**Respondent 123**	5	4	4	3	5
**Respondent 124**	5	5	5	5	5
**Respondent 125**	5	5	5	5	4
**Respondent 126**	5	4	5	5	5
**Respondent 127**	5	4	4	5	5
**Respondent 128**	5	5	5	5	5
**Respondent 129**	5	5	5	5	5
**Respondent 130**	5	4	5	4	5
**Respondent 131**	5	4	4	5	5
**Respondent 132**	5	5	5	5	5
**Respondent 133**	5	5	5	5	4
**Respondent 134**	5	4	5	4	5
**Respondent 135**	5	4	4	3	5
**Respondent 136**	5	5	5	4	5

**Respondent 137**	5	4	5	5	5
**Respondent 138**	5	4	4	5	5
**Respondent 139**	5	5	5	5	4
**Respondent 140**	5	5	5	4	5
**Respondent 141**	5	4	5	5	5
**Respondent 142**	5	4	4	5	5
**Respondent 143**	5	5	5	5	5
**Respondent 144**	5	5	5	5	5
**Respondent 145**	5	4	5	4	4
**Respondent 146**	5	4	4	3	5
**Respondent 147**	5	5	5	4	5
**Respondent 148**	5	5	5	5	5
**Respondent 149**	5	4	5	5	4
**Respondent 150**	5	4	4	5	4
**Respondent 151**	5	5	5	5	5
**Respondent 152**	5	5	5	5	4
**Respondent 153**	5	4	5	4	5
**Respondent 154**	5	4	4	4	5
**Respondent 155**	5	5	5	5	5
**Respondent 156**	5	5	5	5	5
**Respondent 157**	5	4	5	5	5
**Respondent 158**	5	4	4	4	5
**Respondent 159**	5	5	5	5	5

**Respondent 160**	5	5	5	5	5
**Respondent 161**	5	4	5	5	5
**Respondent 162**	5	4	4	5	5
**Respondent 163**	5	5	5	5	5
**Respondent 164**	5	5	5	5	5
**Respondent 165**	5	4	5	5	5
**Respondent 166**	5	4	4	4	3
**Respondent 167**	5	5	5	4	3
**Respondent 168**	5	5	5	5	5
**Respondent 169**	5	4	5	5	3
**Respondent 170**	5	4	4	5	4
**Respondent 171**	5	5	5	5	4
**Respondent 172**	5	5	5	5	4
**Respondent 173**	5	4	5	5	5
**Respondent 174**	5	4	4	4	3
**Respondent 175**	5	5	5	4	3
**Respondent 176**	5	5	5	5	3
**Respondent 177**	5	4	5	4	3
**Respondent 178**	5	4	4	3	5
**Respondent 179**	5	5	5	4	5
**Respondent 180**	5	5	5	5	5
**Respondent 181**	5	4	5	5	4
**Respondent 182**	5	4	4	5	5

**Respondent 183**	5	5	5	4	5
**Respondent 184**	5	5	5	4	5
**Respondent 185**	5	4	5	4	4
**Respondent 186**	5	4	4	4	5
**Respondent 187**	5	5	5	5	5
**Respondent 188**	5	5	5	5	5
**Respondent 189**	5	4	5	5	4
**Respondent 190**	5	4	4	5	5
**Respondent 191**	5	5	5	4	4
**Respondent 192**	5	5	5	5	5
**Respondent 193**	5	4	5	5	4
**Respondent 194**	5	4	4	3	4
**Respondent 195**	5	5	5	4	4
**Respondent 196**	5	5	5	5	4
**Respondent 197**	5	4	5	4	3
**Respondent 198**	5	4	4	5	5
**Respondent 199**	5	5	5	5	5
**Respondent 200**	5	5	5	5	5
**Respondent 201**	5	4	5	5	4
**Respondent 202**	4	4	4	5	4
**Respondent 203**	5	5	5	5	4
**Respondent 204**	3	5	5	5	4
**Respondent 205**	3	4	5	5	5

**Respondent 206**	3	4	4	4	5
**Respondent 207**	5	5	5	5	5
**Respondent 208**	5	5	5	5	5
**Respondent 209**	5	4	5	5	5
**Respondent 210**	5	4	4	4	5
**Respondent 211**	5	5	5	5	5
**Respondent 212**	5	5	5	5	5
**Respondent 213**	5	4	5	5	5
**Respondent 214**	5	4	4	4	5
**Respondent 215**	5	5	5	5	5
**Respondent 216**	5	5	5	5	5
**Respondent 217**	5	4	5	5	5
**Respondent 218**	5	4	4	5	5
**Respondent 219**	5	5	5	5	5
**Respondent 220**	5	5	5	4	5
**Respondent 221**	5	4	5	5	5
**Respondent 222**	5	4	4	4	5
**Respondent 223**	5	5	5	4	5
**Respondent 224**	5	5	5	4	5
**Respondent 225**	5	4	5	4	5
**Respondent 226**	5	4	4	4	5
**Respondent 227**	5	5	5	4	5
**Respondent 228**	5	5	5	5	5

**Respondent 229**	5	4	5	5	5
**Respondent 230**	5	4	4	5	5
**Respondent 231**	5	5	5	5	5
**Respondent 232**	3	5	5	4	5
**Respondent 233**	5	4	5	5	5
**Respondent 234**	5	4	4	5	5
**Respondent 235**	5	5	5	5	5
**Respondent 236**	5	5	5	5	5
**Respondent 237**	5	4	5	5	5
**Respondent 238**	5	4	4	5	5
**Respondent 239**	5	5	5	4	5
**Respondent 240**	5	5	5	3	5
**Respondent 241**	5	4	5	5	5
**Respondent 242**	3	4	4	5	5
**Respondent 243**	5	5	5	5	5
**Respondent 244**	5	5	5	5	5
**Respondent 245**	5	4	5	4	5
**Respondent 246**	5	4	4	4	5
**Respondent 247**	5	5	5	4	5
**Respondent 248**	5	5	5	4	5
**Respondent 249**	5	4	5	5	5
**Respondent 250**	5	4	4	5	5
**Respondent 251**	5	5	5	5	5

**Respondent 252**	5	5	5	5	5
**Respondent 253**	5	4	5	4	5
**Respondent 254**	5	4	4	5	5
**Respondent 255**	3	5	5	5	5
**Respondent 256**	5	5	5	4	5
**Respondent 257**	5	4	5	4	5
**Respondent 258**	5	4	4	4	5
**Respondent 259**	5	5	5	4	5
**Respondent 260**	5	5	5	4	5
**Respondent 261**	5	4	5	4	5
**Respondent 262**	5	4	4	4	5
**Respondent 263**	5	5	5	4	5
**Respondent 264**	5	5	5	5	5
**Respondent 265**	5	4	5	5	5
**Respondent 266**	5	4	4	5	5
**Respondent 267**	5	5	5	5	5
**Respondent 268**	3	5	5	4	5
**Respondent 269**	5	4	5	4	5
**Respondent 270**	5	4	4	4	5
**Respondent 271**	5	5	5	4	5
**Respondent 272**	5	5	5	4	5
**Respondent 273**	5	4	5	4	5
**Respondent 274**	5	4	4	5	3

**Respondent 275**	5	5	5	5	3
**Respondent 276**	5	5	5	4	4
**Respondent 277**	5	4	5	4	5
**Respondent 278**	5	4	4	3	4
**Respondent 279**	5	5	5	4	4
**Respondent 280**	5	5	5	5	4
**Respondent 281**	5	4	5	4	3
**Respondent 282**	3	4	4	5	3
**Respondent 283**	3	5	5	5	3
**Respondent 284**	5	5	5	5	5
**Respondent 285**	5	4	5	5	3
**Respondent 286**	5	4	4	5	5
**Respondent 287**	5	5	5	5	5
**Respondent 288**	5	5	5	5	5
**Respondent 289**	5	4	5	4	4
**Respondent 290**	5	4	4	4	4
**Respondent 291**	5	5	5	5	4
**Respondent 292**	5	5	5	5	4
**Respondent 293**	5	4	5	5	4
**Respondent 294**	5	4	4	4	5
**Respondent 295**	5	5	5	4	4
**Respondent 296**	5	5	5	4	5
**Respondent 297**	5	4	5	4	5

**Respondent 298**	5	4	4	4	5
**Respondent 299**	5	5	5	4	5
**Respondent 300**	5	5	5	5	5
**Respondent 301**	5	4	5	5	5
**Respondent 302**	5	4	4	4	5
**Respondent 303**	5	5	5	4	5
**Respondent 304**	5	5	5	4	5
**Respondent 305**	5	4	5	4	5
**Respondent 306**	5	4	4	5	4
**Respondent 307**	5	5	5	5	4
**Respondent 308**	5	5	5	5	4
**Respondent 309**	5	4	5	5	5
**Respondent 310**	5	4	4	5	5
**Respondent 311**	5	5	5	4	4
**Respondent 312**	5	5	5	5	4
**Respondent 313**	5	4	5	5	5
**Respondent 314**	5	4	4	5	4
**Respondent 315**	5	5	5	3	5
**Respondent 316**	5	5	5	4	4
**Respondent 317**	5	4	5	3	3
**Respondent 318**	5	4	4	5	5
**Respondent 319**	5	5	5	5	5
**Respondent 320**	5	5	5	5	5

**Respondent 321**	5	4	5	5	3
**Respondent 322**	5	4	4	4	5
**Respondent 323**	5	5	5	5	4
**Respondent 324**	5	5	5	5	5
**Respondent 325**	4	4	5	5	5
**Respondent 326**	5	4	4	5	4
**Respondent 327**	4	5	5	5	5
**Respondent 328**	4	5	5	3	5
**Respondent 329**	5	4	5	4	4
**Respondent 330**	4	4	4	3	5
**Respondent 331**	4	5	5	5	5
**Respondent 332**	5	5	5	5	3
**Respondent 333**	5	4	5	3	5
**Respondent 334**	5	4	4	5	3
**Respondent 335**	4	5	5	4	5
**Respondent 336**	5	5	5	5	3
**Respondent 337**	5	4	5	5	5
**Respondent 338**	4	4	4	5	5
**Respondent 339**	5	5	5	5	4
**Respondent 340**	5	5	5	4	5
**Respondent 341**	5	4	5	5	5
**Respondent 342**	5	4	4	5	3
**Respondent 343**	5	5	5	5	5

**Respondent 344**	3	5	5	5	5
**Respondent 345**	4	4	5	4	5
**Respondent 346**	3	4	4	5	4
**Respondent 347**	2	5	5	5	5
**Respondent 348**	3	5	5	5	5
**Respondent 349**	3	4	5	5	5
**Respondent 350**	4	4	4	5	3
**Respondent 351**	4	5	5	4	5
**Respondent 352**	3	5	5	5	5
**Respondent 353**	4	4	5	5	5
**Respondent 354**	3	4	4	5	4
**Respondent 355**	4	5	5	4	5
**Respondent 356**	3	5	5	5	5
**Respondent 357**	3	4	5	4	5
**Respondent 358**	5	4	4	5	5
**Respondent 359**	5	5	5	5	3
**Respondent 360**	4	5	5	5	5
**Respondent 361**	5	4	5	4	5
**Respondent 362**	5	4	4	4	5
**Respondent 363**	5	5	5	5	4
**Respondent 364**	5	5	5	5	5
**Respondent 365**	5	4	5	5	5
**Respondent 366**	5	4	4	5	5

**Respondent 367**	5	5	5	5	3
**Respondent 368**	5	5	5	5	5
**Respondent 369**	5	4	5	5	5
**Respondent 370**	5	4	4	5	5
**Respondent 371**	5	5	5	5	5
**Respondent 372**	5	5	5	5	4
**Respondent 373**	5	4	5	5	5
**Respondent 374**	5	4	4	5	5
**Respondent 375**	5	5	5	5	5
**Respondent 376**	5	5	5	5	3
**Respondent 377**	5	4	5	5	5
**Respondent 378**	5	4	4	4	5
**Respondent 379**	5	5	5	5	4
**Respondent 380**	5	5	5	5	5
**Respondent 381**	5	4	5	5	5
**Respondent 382**	5	4	4	5	5
**Respondent 383**	5	5	5	5	5
**Respondent 384**	5	5	5	5	5
**Respondent 385**	5	4	5	5	3
**Respondent 386**	5	4	4	5	4
**Respondent 387**	5	5	5	4	5
**Respondent 388**	5	5	5	5	5
**Respondent 389**	5	4	5	3	5

**Respondent 390**	5	4	4	5	4
**Respondent 391**	5	5	5	5	5
**Respondent 392**	5	5	5	5	5
**Respondent 393**	5	4	5	5	5
**Respondent 394**	5	4	4	5	5
**Respondent 395**	5	5	5	5	4
**Respondent 396**	5	5	5	5	5
**Respondent 397**	5	4	5	5	5
**Respondent 398**	5	4	4	5	5
**Respondent 399**	5	5	5	4	5
**Respondent 400**	5	5	5	5	3
**Respondent 401**	5	4	5	5	5
**Respondent 402**	5	4	4	5	5
**Respondent 403**	4	5	5	5	5
**Respondent 404**	4	5	5	5	3
**Respondent 405**	4	4	5	4	5
**Respondent 406**	4	4	4	5	5
**Respondent 407**	4	5	5	4	5
**Respondent 408**	4	5	5	5	3
**Respondent 409**	4	4	5	3	5
**Respondent 410**	4	4	4	5	5
**Respondent 411**	4	5	5	4	5
**Respondent 412**	4	5	5	5	5

**Respondent 413**	4	4	5	5	5
**Respondent 414**	4	4	4	5	5
**Respondent 415**	4	5	5	5	3
**Respondent 416**	4	5	5	4	5
**Respondent 417**	5	4	5	5	3
**Respondent 418**	5	4	4	5	5
**Respondent 419**	3	5	5	5	5
**Respondent 420**	3	5	5	5	3
**Respondent 421**	5	4	5	5	5
**Respondent 422**	3	4	4	5	5
**Respondent 423**	3	5	5	5	5
**Respondent 424**	3	5	5	5	5
**Respondent 425**	3	4	5	5	3
**Respondent 426**	5	4	4	5	5
**Respondent 427**	5	5	5	5	5
**Respondent 428**	4	5	5	5	5
**Respondent 429**	3	4	5	5	5
**Respondent 430**	5	4	4	4	5
**Respondent 431**	5	5	5	5	5
**Respondent 432**	3	5	5	5	5
**Respondent 433**	5	4	5	5	5
**Respondent 434**	5	4	4	5	5
**Respondent 435**	5	5	5	5	5
